# Anti-Rift Valley fever virus activity *in vitro*, pre-clinical pharmacokinetics and oral bioavailability of benzavir-2, a broad-acting antiviral compound

**DOI:** 10.1038/s41598-018-20362-9

**Published:** 2018-01-31

**Authors:** Md. Koushikul Islam, Mårten Strand, Michael Saleeb, Richard Svensson, Pawel Baranczewski, Per Artursson, Göran Wadell, Clas Ahlm, Mikael Elofsson, Magnus Evander

**Affiliations:** 1 Department of Clinical Microbiology, Infectious Diseases, Umeå University, Umeå, Sweden; 2 Department of Clinical Microbiology, Virology, Umeå University, Umeå, Sweden; 3 Department of Chemistry, Umeå University, Umeå, Sweden; 4 Uppsala Drug Optimization and Pharmaceutical Profiling Platform (UDOPP), Department of Pharmacy, Uppsala University, Uppsala, Sweden; 5 SciLifeLab Drug Discovery and Development Platform, ADME of Therapeutics, Department of Pharmacy, Uppsala University, Uppsala, Sweden; 6 Department of Drug Delivery, Institute of Pharmacy, Uppsala University, Uppsala, Sweden

## Abstract

Rift Valley fever virus (RVFV) is a mosquito-borne hemorrhagic fever virus affecting both humans and animals with severe morbidity and mortality and is classified as a potential bioterror agent due to the possible aerosol transmission. At present there is no human vaccine or antiviral therapy available. Thus, there is a great need to develop new antivirals for treatment of RVFV infections. Benzavir-2 was previously identified as potent inhibitor of human adenovirus, herpes simplex virus type 1, and type 2. Here we assess the anti-RVFV activity of benzavir-2 together with four structural analogs and determine pre-clinical pharmacokinetic parameters of benzavir-2. *In vitro*, benzavir-2 efficiently inhibited RVFV infection, viral RNA production and production of progeny viruses. *In vitro*, benzavir-2 displayed satisfactory solubility, good permeability and metabolic stability. In mice, benzavir-2 displayed oral bioavailability with adequate maximum serum concentration. Oral administration of benzavir-2 formulated in peanut butter pellets gave high systemic exposure without any observed toxicity in mice. To summarize, our data demonstrated potent anti-RVFV activity of benzavir-2 *in vitro* together with a promising pre-clinical pharmacokinetic profile. This data support further exploration of the antiviral activity of benzavir-2 in *in vivo* efficacy models that may lead to further drug development for human use.

## Introduction

Rift Valley fever virus (RVFV), a negative-stranded RNA virus from the *Phenuiviridae* family, *Phlebovirus* genus, is the etiological agent of the zoonotic disease Rift Valley fever (RVF). RVFV is an emerging virus that causes significant morbidity and mortality in humans and livestock throughout Africa and Arabian Peninsula. RVFV is capable of infecting a broad range of mosquito species found around the globe and therefore has the potential to spread to the other parts of the world. In humans RVFV infection presents as an acute self-limiting febrile illness, but severe manifestations, including hemorrhagic fever and encephalitis, also occur, with case fatality rates >30% reported in some outbreaks, and long-term sequelae (e.g. impaired vision) may occur in survivors^1–3^. Recently, we have shown for the first time an association of RVFV infection with miscarriage in humans^4^. In pregnant ruminants the virus causes abortion storms with >90% mortality rate. Young animals have high mortality rates while adult animals are less susceptible to RVFV^5^. Moreover, RVFV can be transmitted by aerosol exposure and cause severe encephalitis in a murine model^6^. RVFV is classified as a potential bioterror agent due to the possible aerosol transmission and the severe disease caused by the virus^7^. Importantly, no licensed vaccines or effective antiviral therapies are available to treat RVFV disease. Vaccines used in livestock have major safety concerns and there are no conclusive reports on antiviral drugs used in clinical settings for the treatment of RVFV^8,9^. Ribavirin, Favipiravir and curcumin have shown anti-RVFV activity *in vitro* and in animal models but have not been assessed in clinical trials against RVFV^10–12^. Thus, there is a great need to develop new antivirals for treatment of RVFV infections.

We have previously identified benzavir-1 as an antiviral compound against HAdV using a whole-cell based screening assay^13^. Subsequently, benzavir-1 was optimized to benzavir-2, with improved efficacy against HAdV and lower host cell toxicity by structure-activity relationship analysis^14^. Benzavir-2 is a novel compound that efficiently inhibits both human adenovirus (HAdV)^13^, herpes simplex virus type 1 and type 2 (HSV-1, HSV-2)^15^. We have also shown that benzavir-2 can inhibit clinical acyclovir-resistant isolates of HSV-1 and HSV-2^15^. Here we are addressing the efficacy of benzavir-2 and structural analogs against RVFV infection, assess the pre-clinical pharmacokinetic (PK) parameters and demonstrate that high systemic exposure *in vivo* can be achieved after oral administration of benzavir-2 when formulated in peanut butter pellets. Furthermore, we discuss the potential of benzavir-2 as a starting-point for further drug development for human use.

## Results

### Benzavir-2 efficiently inhibited RVFV infection

**Figure 1:**
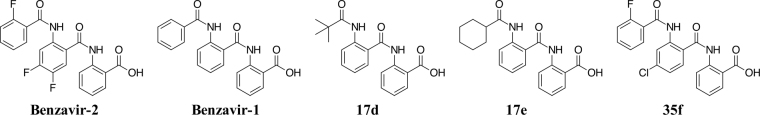
Structure of benzavir-2, benzavir-1, 17d, 17e and 35f.

**Figure 2:**
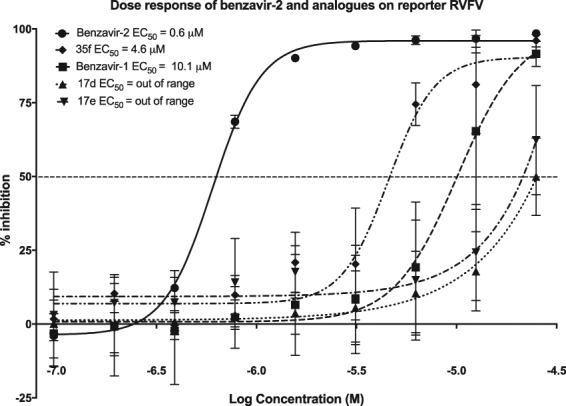
Dose-response curves of benzavir-2, benzavir-1, 17d, 17e, 35f. The inhibitory effect of compounds on the fluorescent intensity of rRVFVΔNSs::Katushka-infected A549 cells in comparison to untreated cells. Compound concentrations ranged from 25 μM to 0.097 μM in two-fold dilutions. The EC_50_ values were calculated based on three independent experiments with two replicates each time.

**Figure 3:**
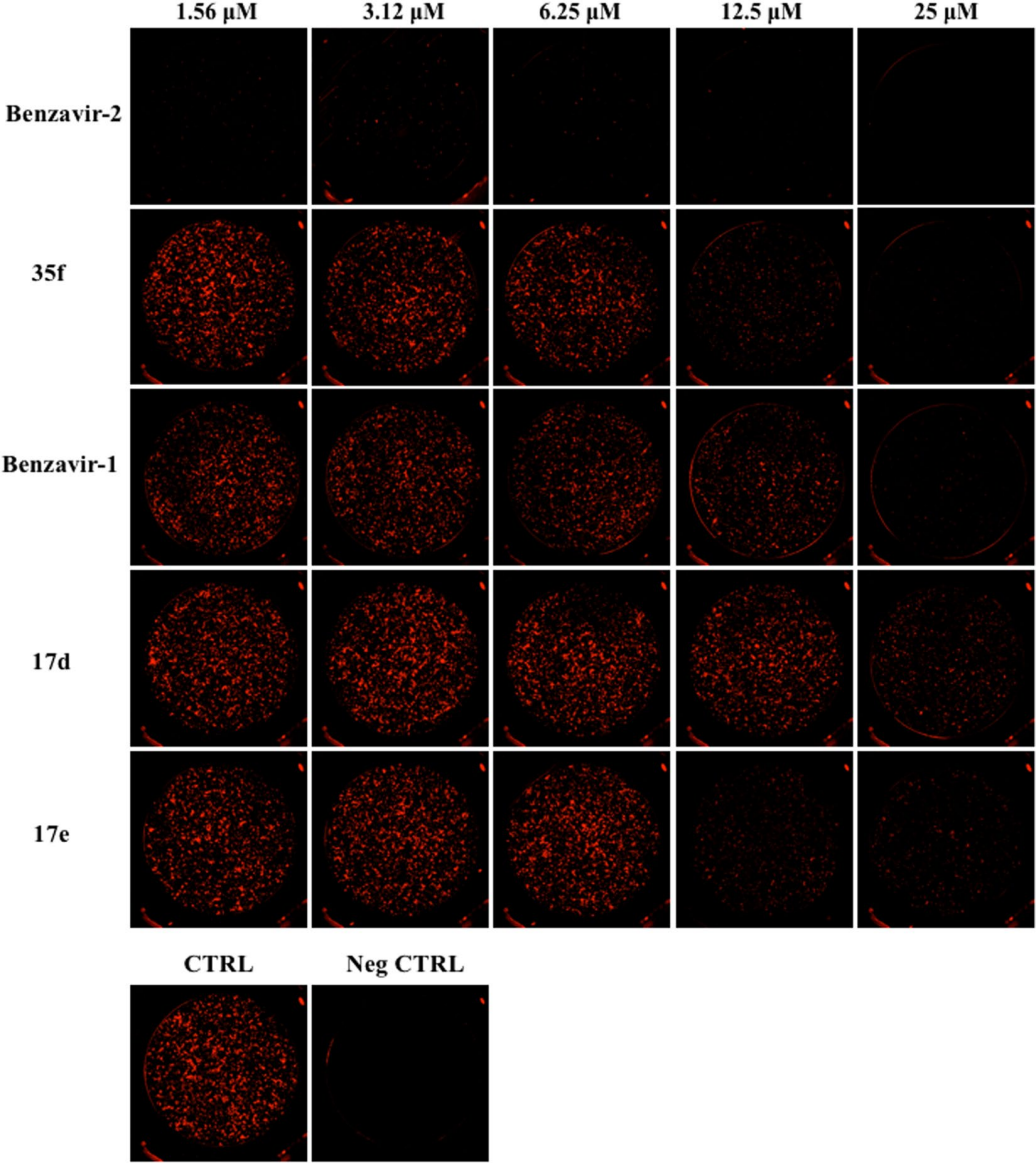
Immunofluorescence of rRVFVΔNSs::Katushka-infected cells. A549 cells were infected and treated with benzavir-2, 35f, benzavir-1, 17d, 17e. CTRL is virus infected cells with no treatment. Neg CTRL is only cells. Five of the inhibitory compound concentrations are displayed.

**Figure 4:**
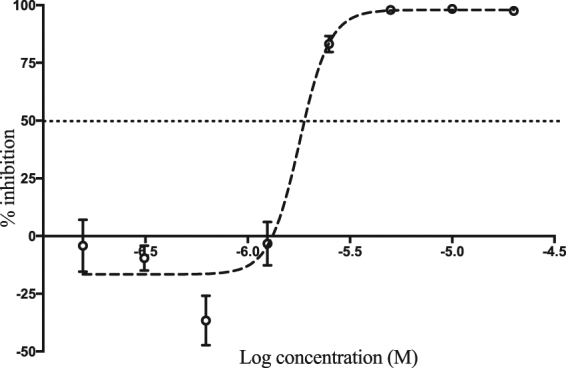
Inhibition of viral RNA expression by benzavir-2. The inhibitory effect of benzavir-2 on viral RNA expression 16 h post infection was quantified in rRVFVΔNSs::Katushka infected A549 cells in comparison to untreated cells. The benzavir-2 concentrations ranged from 20 μM to 0.156 μM. The EC_50_ value (1.7 μM) was calculated based on three independent experiments with two replicates each time.

To address the potential anti-RVFV activity of benzavir-2 and structural analogs (Fig. 1) we assessed the inhibition of expression of a far-red fluorescent reporter protein (Katushka) expressed after infection of the recombinant RVFV (rRVFVΔNSs::Katushka). In this recombinant infectious virus, the RVFV non-structural gene NSs is deleted and replaced by the Katushka reporter gene, which is expressed after infection. Using a fluorescence cell foci assay we could observe, as in previous studies, that benzavir-2 displayed the highest potency among the five analogs with an EC_50_ value 0.6 μM^14^. Benzavir-1 and compound 35f were less efficient with an EC_50_ value of 10.1 μM and 4.5 μM respectively (Fig. 2). No accurate EC_50_ value for compound 17d and 17e could be obtained due to low activity as illustrated in Fig. 3. Interestingly, this trend in potency against RVFV follows the structure-activity relationships previously observed when assessed against HAdV^14^. Moreover, the sigmoidal dose-response curves, preferably seen for benzavir-2 (Fig. 2), has previously been observed against both HAdV and HSV. Since benzavir-2 displayed the most potent antiviral activity, we decided to focus on benzavir-2 in the succeeding assessments. To further explore the anti-RVFV effect of benzavir-2 observed using the fluorescence cell foci assay, we utilized quantitative real-time PCR (qPCR) to measure the potential inhibitory effect of benzavir-2 on viral RNA expression. Cells were infected with rRVFVΔNSs::Katushka for 16 h, total cellular RNA was extracted, and levels of RVFV RNA were measured by qPCR. The results showed a dose-dependent inhibition of RVFV RNA expression by benzavir-2 (Fig. 4) with an EC_50_ value of 1.7 μM.

### Benzavir-2 inhibited the production of progeny RVFV

**Figure 5:**
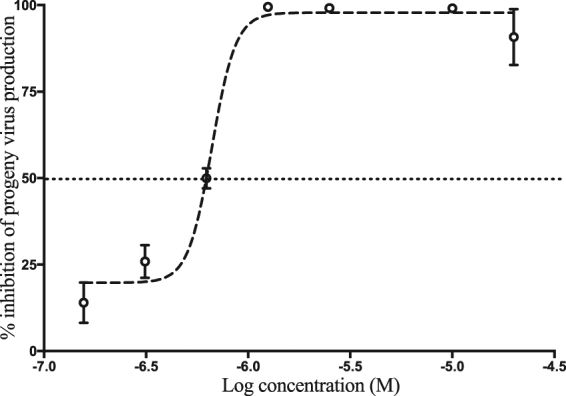
Inhibition of progeny virus production by benzavir-2. The supernatants from rRVFVΔNSs::Katushka infected cells treated with different concentrations (20 μM to 0.156 μM) of benzavir-2 were collected. Then, A549 cells were infected with the different supernatants for 16 h to determine the inhibition of production of infectious progeny virus. The number of infected cells were counted by fluorescent foci. The EC_50_ value (0.6 μM) was calculated based on two independent experiments with six replicates each time.

We next analyzed whether the benzavir-2 inhibition of infected cell foci and viral RNA expression also affected the production of infectious progeny virus. For this, supernatants from rRVFVΔNSs::Katushka infected cells were collected and used to infect new cell layers. The infection was measured using the fluorescence cell foci assay, and benzavir-2 efficiently inhibited the production of progeny infectious virus in a dose-dependent manner (Fig. 5) with an EC_50_ value of 0.6 μM.

### Time-of-addition study

**Figure 6:**
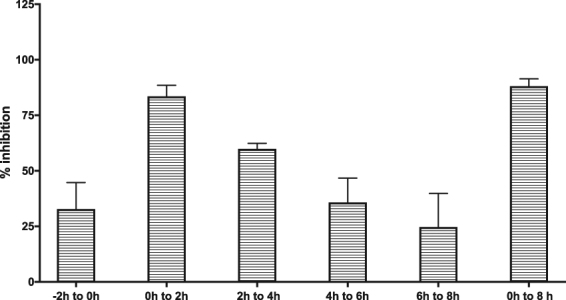
Time-of-addition experiment. Benzavir-2, at a concentration of 20 μM, was added to A549 cells in 2 h intervals starting 2 h prior infection with rRVFVΔNSs::Katushka. Analysis was performed 8 h post infection using the fluorescent cell foci assay.

In order to obtain insight into the putative mode-of-action of benzavir-2, benzavir-2 was added at different time points during the virus infection. Based on the growth curve of rRVFVΔNSs::Katushka, the read-out was performed 8 h postinfection when virus titers were in the logarithmic growth phase. When benzavir-2 (20 μM) was added early in the infection cycle, the inhibitory effect on the formation of the fluorescent cell foci was the greatest. Addition of benzavir-2 at later time-points post infection had less inhibition. In addition, pre-incubation of the cells with benzavir-2 prior infection had less inhibitory effect compared to addition of benzavir-2 in the early phase of infection (Fig. 6).

### Toxicity of benzavir-2 on host cells

The toxicity of benzavir-2 and its analogs have been addressed in previous publications^13–15^. Benzavir-2 has been assessed on both human lung epithelial cells (A549) and Green monkey kidney cells (GMK) using XTT-based toxicity test, a colorimetric assay to distinguish metabolically active cells. Due to solubility issues of benzavir-2 when exceeding 150 μM in cell media (unpublished data) we set the concentration for toxicity assessment to 30—and 60 μM. On A549 cells, which is used in this study, the percentage of viable cells were 96% at both concentrations^14^. Hence, the CC_50_ value was not achieved within these concentrations and no Selectivity Index (SI) for benzavir-2 could be presented.

### *In vitro* preclinical profiling benzavir- 1 and benzavir-2

The *in vitro* absorption, distribution, metabolism and excretion (ADME) data acquired for the most interesting compounds are shown in Table 1. Certain data are lacking from benzavir-1 since throughout this study more focus was put on the more efficacious benzavir-2. As expected, the two compounds behaved similarly in the different ADME assays. A notable difference was the higher apparent solubility at pH 7.4 of benzavir-1, which was not reflected in the measured distribution coefficient (LogD) (Table 1). The compounds were metabolically stable in the presence of both human and mouse liver microsomes and hepatocytes, and had high permeabilities in Caco-2 cell monolayers (Table 1). The efflux ratio was close to one, indicating lack of cellular resistance due to efflux mechanisms (Table 1). Scaled clearance (CL) in both human hepatocytes and liver microsomes gave comparable values (unbound scaled hepatic clearance, CL_H, u_) (Table 1), indicating a limited impact of hepatocyte glucuronidation or sulphation^16,17^. Both compounds bound to a high level to plasma proteins (Table 1).

**Table 1:** *In vitro* ADME properties.

*In vitro* ADME-parameters	Benzavir-1	Benzavir-2
MW (g/mol)	360.4	414.3
Log D	1.52 ± 0.02	1.56 ± 0.01
cLog P	4.75	5.52
Solubility pH 7.4 (mM)	0.93 ± 0.11	0.13 ± 0.01
Fraction unbound (human, %)	0.03	0.04 ± 0.02
Fraction unbound (mouse, %)	n.d.	0.76 ± 0.02
Caco-2 Papp (a-b) (×10^6^ cm/s)	32 ± 3	55 ± 4
Caco-2 Papp (b-a) (×10^6^ cm/s)	31 ± 2	45 ± 3
Efflux ratio	1.0	0.8
CL_int_ [μl/mg/min] (HLM)	3.9 ± 0.8	3.8 ± 0.4
CL_H, u_ [l/h] (HLM)	1.0	1.4
CL_int_ [μl/min/10^6^ cells] (HHep)	3.1 ± 0.1	4.1 ± 0.4
CL_H, u_ [l/h] (HHep)	2.3	4.0
CL_int_ [μl/mg/min] (MLM)	n.d.	3.8 ± 0.9
CL_H, u_ [l/h] (MLM)	n.d.	0.15

Abbreviations: ADME = absorption, distribution, metabolism and excretion, MW = molecular weight, Log D = logarithmic ratio of distribution coefficient, cLog P = calculated logarithmic ratio of partition coefficient, Papp = apparent permeability coefficient, a-b = apical to basolateral, b-a = basolateral to apical, CL = clearance, CLint = intrinsic CL, CLH = hepatic CL, u = unbound, HLM = human liver microsomes, MLM = mouse liver microsomes, HHep = fresh human hepatocytes, n.d. = not determined.

### *In vivo* pharmacokinetics of benzavir-2

**Figure 7:**
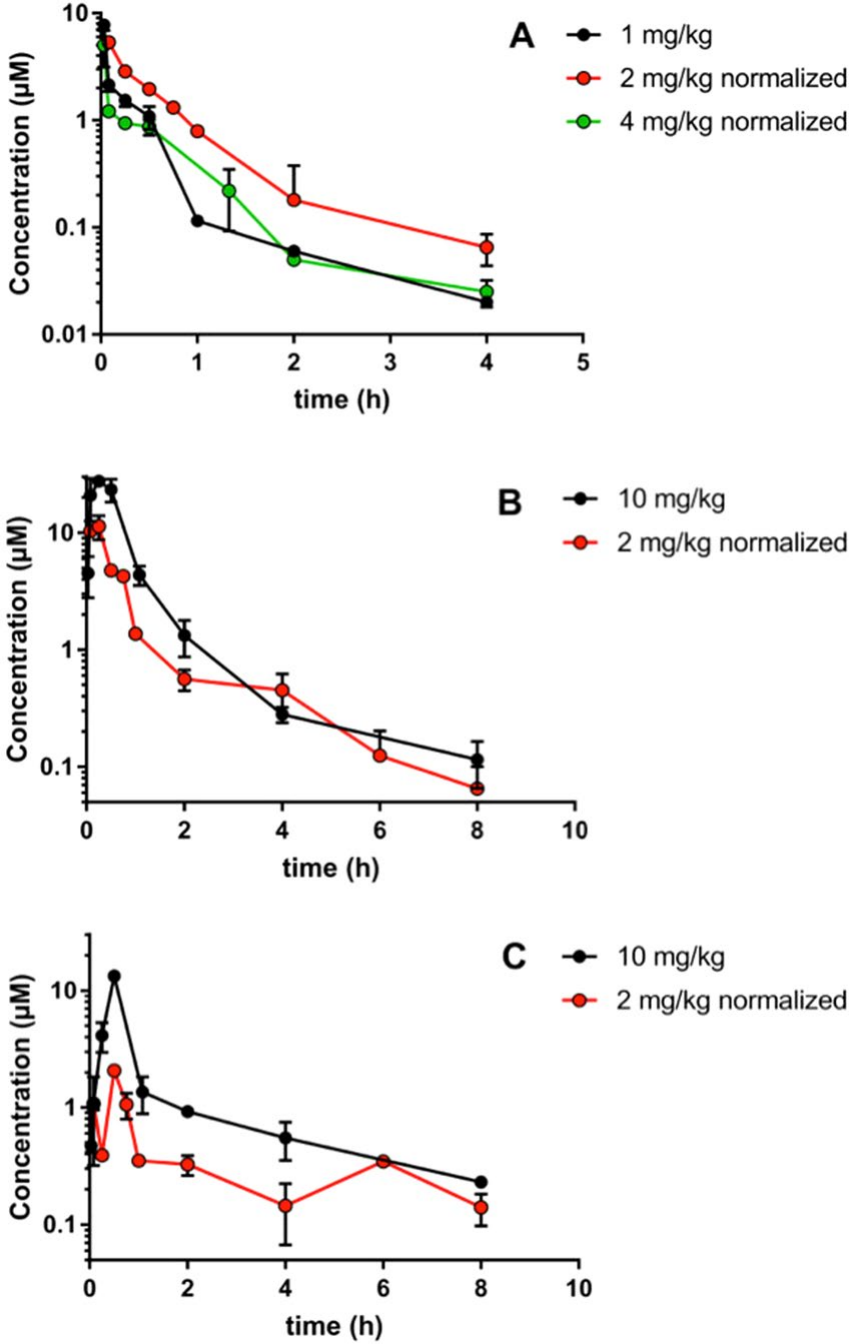
Log-linear plots of benzavir-2 pharmacokinetics in mice. Normalized plasma concentration data to intravenous 1 mg/kg, intraperitoneal and orally by oral gavage (po) 10 mg/kg. (**A**) iv administration. (**B**) ip administration. (**C**) po administration. n = 2 per time point, error bars indicate SD. Formulation 1 was used for the 2 mg/kg dose for all administration routes and for the 1 mg/kg iv administration. Formulation 2 was used for 10 mg/kg ip and po administration and for the 4 mg/kg iv administration.

The PK properties of benzavir-2, which displayed promising *in vitro* antiviral and ADME properties as described above, was investigated in mice. A pilot iv, ip and po study of benzavir-2 formulated in liquid formulation indicated very rapid extravascular absorption (not shown). This motivated blood sampling at early time points in the proceeding studies also for po to accurately monitor the PK. Figure 7 shows the results of two repeated PK experiments, using different routes of administration using liquid formulation. The data were normalized to the indicated dose for easier comparison of the PK parameters. The samples retrieved after iv administration showed distinct biphasic behavior, a clear disposition and elimination, and fairly good linear correlation on the dose normalization (Fig. 7). The calculated volume of distribution (Vd) indicated considerable tissue distribution. The iv CL showed slight variation but did not differ between doses, suggesting that the elimination routes remained unsaturated in the investigated dose interval. In comparison to the unbound *in vitro* metabolic CL, the iv CL was 2–4 times higher which may indicate additional, non-hepatic, elimination mechanisms.

In contrast, the extravascular PK showed poor linear correlations between different doses. The calculated bioavailability (F_app_) (normalized) increased around 2 and 4-fold for ip and po, respectively when the dose was increased from 2 to 10 mg/kg. This suggest saturation of metabolic pathways in the intestine and liver first-pass. The 2–4-fold increased elimination half-life after ip and po, respectively, as compared to iv administration, suggests that absorptive and/ or dissolution processes are affected by physiological differences between the administration routes, which will influence the terminal elimination.

### Benzavir-2 formulated in peanut butter (PB) pellets yields high systemic exposure

**Figure 8:**
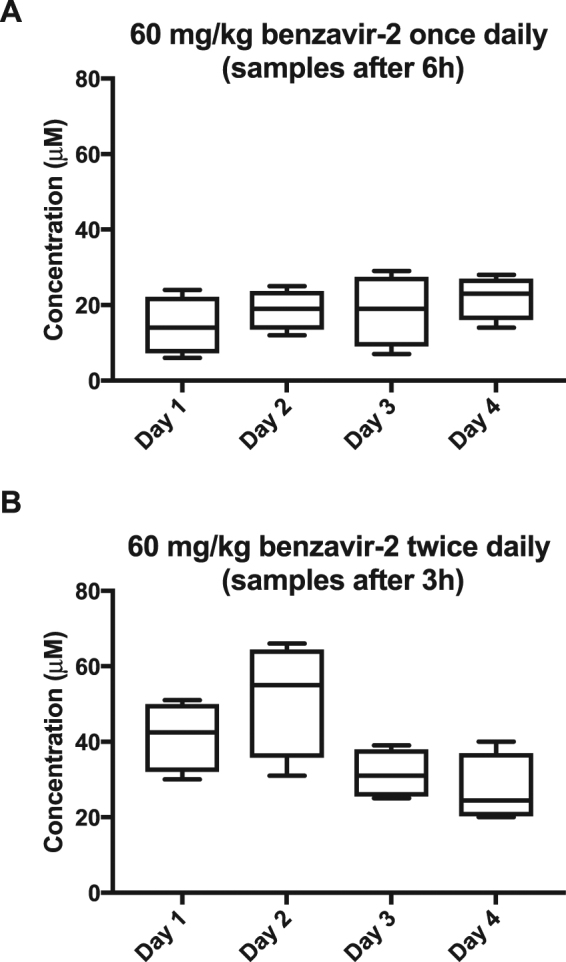
Plasma concentration of benzavir-2 formulated in peanut butter pellets. Oral administration of peanut butter pellets containing 60 mg/kg benzavir-2 once daily with blood sample after 6 h (**A**) and twice daily with 6 h interval and blood sample 3 h after first administration (**B**). (**A**,**B**) display the plasma concentration of benzavir-2 in 4 mice over 4 days.

The liquid formulations used for the PK assessments of benzavir-2 *in vivo* limits the maximum dose that can be administered in mouse. Hence, we explored formulation alternatives that potentiates delivery of a significant higher administration dose, in order to assess any negative clinical signs associated with benzavir-2 *in vivo*. One formulation alternative is PB pellets that previously have been used with promising results^18^. The plasma concentrations of benzavir-2 were measured after oral administration of 60 mg/kg once daily for four consecutive days (Fig. 8A) and oral administration of 60 mg/kg twice daily for four consecutive days (Fig. 8B). Note the difference in time-points of blood sampling after the benzavir-2 administration. The mean plasma concentrations of benzavir-2, administered once daily, varied between approximately 15 and 20 μM, when analyzed 6 h after administration (Fig. 8A). When benzavir-2 was administered twice daily, the mean plasma concentration was increased to approximately 50 and 27 μM, analyzed 3 h after administration (Fig. 8B). There were variations between the individual mice that could be explained by occasional incomplete consumption of the PB pellet. No accumulation of benzavir-2 in the plasma over time could be observed and the data indicated that benzavir-2 was eliminated from the plasma between the administration time-points. There was a trend of declining plasma concentrations at day 3 and day 4, that could indicate a change of the pharmacokinetics upon multiple dosing but needs further testing to be verified (Fig. 8). No weight loss or negative behavioural signs were observed in the mice throughout the 4 days.

## Discussion

RVF is a mosquito borne zoonotic disease causing regular outbreaks throughout the African subcontinent with a serious impact on human health and animal productivity. The most recent outbreak was in the Republic of Niger in October 2016 with a case-fatality rate of 31%^19^. It is predicted that it is just a matter of time until RVFV will spread to other parts of the world since *Aedes* and *Culex* mosquitoes, the most common vectors for RVFV, are present globally^20–22^. Previously we have presented the antiviral activity of benzavir-2 against HAdV, HSV-1 and HSV-2 infection *in vitro*^13,15^, all of them are DNA viruses that replicate in the nucleus. Here, we assessed the antiviral activity of benzavir-2 together with four structural analogs on RVFV, an RNA virus with membrane-associated replication that takes place in the cytoplasm.

We utilized the replication-competent rRVFVΔNSs::Katushka vector, classified as a biosafety level 2 pathogen, as a model for the highly pathogenic wild type RVFV. Benzavir-2 displayed potent *in vitro* antiviral activity against rRVFVΔNSs::Katushka infection, similar to the potency against HAdV and HSV. Compared to benzavir-2, benzavir-1 was less potent in our assay with higher EC_50_ value than previously observed against HAdV^13,14^ and HSV^15^. Compound 35f displayed improved potency compared to benzavir-1. In a previous study, compound 35f was less potent than benzavir-1 against HAdV. However, the low potency for compound 17d and 17e, previously seen against HAdV^14^, was also observed against RVFV and the EC_50_ values could not be determined in the analyzed dose-range.

After identifying benzavir-2 as a potent inhibitor of RVFV infection with inhibitory effect on the expression of rRVFVΔNSs::Katushka (EC_50_ = 0.6 µM) (Figs 2 and 3) and the number of produced viral RNA genomes (EC_50_ = 1.7 µM) (Fig. 4), we further evaluated if the inhibition also was reflected on the number of rRVFVΔNSs::Katushka progeny particles. As seen in Fig. 5, the number of viable progeny virus particles released from the infected cells was clearly inhibited with increasing concentrations of benzavir-2 (EC_50_ = 0.6 µM). The inhibition of the production of viral genomes and fluorescent cell foci correlated with the same EC_50_ value (0.6 µM) while the inhibition of viral RNA expression had a slightly higher EC_50_ value (1.7 µM) (Figs 2–4). The viral RNA produced inside the cell does not have to be correlated with virus progeny production. It is likely that more viral RNA is produced than would result in progeny particles. From the results, it is clear that the viral RNA production is inhibited, although at higher EC_50_. In the presence of 1.25 μM of benzavir-2, the release of viable progeny particles to the supernatant was almost completely inhibited (>95%) (Fig. 5). This indicated that benzavir-2 not only inhibited the expression and replication of rRVFVΔNSs::Katushka, but also potently inhibited the cell-to-cell spread of the virus. From an antiviral drug development perspective, the capacity to efficiently reduce and almost block the viral spread is attractive.

The mechanism-of-action and protein target(s) for benzavir-2 are currently not known. However, considering that benzavir-2 previously has been reported to have activity against HAdV and HSV which both are DNA viruses that replicates in the nucleus, our data presented here demonstrated that benzavir-2 has a broad-acting antiviral activity. RVFV is a RNA virus that replicates in the cytoplasm and most probably there are a limited number of proteins shared with HAdV and HSV during their infectious cycle that can act as targets for benzavir-2. This may indicate that benzavir-2 acts on a host cell target. Moreover, the ranking of the five compounds tested in this study is similar to the ranking based on inhibition of HAdV and the inhibition follows the previously seen sigmoidal shaped dose-response curves^15^. This may indicate that the target(s) responsible for the antiviral activity is the same for HAdV, rRVFVΔNSs::Katushka and perhaps also for HSV. From a drug-resistance point of view, inhibition of a host cell target may be beneficial^23^. On the other hand, activity against a host cell target may be associated with host cell toxicity, but this is not observed by benzavir-2 at relevant concentrations^13–15^. Studies to identify the mechanism-of-action for benzavir-2 are ongoing and the time-of addition analysis we performed was a first step. The results indicated that benzavir-2 was most potent early in the infection and was reduced at later stages. No inhibition of binding or uptake of RVFV virions by benzavir-2 has been observed (data not shown), indicating that benzavir-2 acts on an intracellular target that is essential in the early phase of the RVFV infection (e.g. transcription and/or translation). We currently pursue the isolation of resistant mutants, which could guide us to understand the precise mechanism of action for benzavir-2.

To elucidate if benzavir-2 has the potential to be further assessed in efficacy models, we determined PK properties of benzavir-2 by performing *in vitro* ADME and *in vivo* PK studies in mouse. Moreover, we show that a high systemic exposure of benzavir-2 can be reached after oral administration in PB pellets without any observed toxicity in mice. The *in vitro* ADME studies indicated that benzavir-2 displayed many advantageous PK properties, including good solubility and permeability and moderate metabolic stability. The high plasma protein binding (Table 1) did not limit the tissue distribution, suggesting rapid on-off kinetics (Table 2). The PK studies in mice showed promising properties of benzavir-2, with good oral bioavailability and satisfactory C_max_ and bioavailability parameters. The relatively fast T_max_ parameter suggests a rapid uptake from all administration routes. The elimination half-life (T_1/2_) and the F_app_ however differed between administration routes that suggests saturation of metabolism during first-passage over intestine and/or liver. The increase of T_1/2_ between ip and po also indicates that the intestinal absorption may be affected either by saturation of transport and/or altered dissolution rate. Although benzavir-2 show high Caco-2 permeability we currently do not know to what extent the transport is active and passive, respectively. The solubility of benzavir-2 is moderate while metabolism appears saturable, which proposes that it could belong to the BDDCS cla**s**ses applicable to flip-flop kinetics as discussed in Garrison *et al*.^24^. Further, the apparent nonlinear kinetics indicated that high oral doses may be administered, resulting in a good systemic exposure. An outstanding issue is the complex extravascular pharmacokinetics of benzavir-2, which will require further studies to unravel. Taken together, the ADME and PK studies indicated that benzavir-2 has good drug-like properties and shows promise in comparison to existing antiviral drugs, such as acyclovir, in terms of e.g. permeability^25^ and oral bioavailability^26^.

**Table 2:** Mouse *in vivo* pharmacokinetic parameters.

	IV			IP		PO	
Dose (mg/kg)	1	2	4	2	10	2	10
Cmax (µM)	7.8	7.8	20	2.3	28	0.4	13
Tmax (min)	—	—	—	15	15	30	30
AUC (µM×h) (normalized)	1.8 ± 0.1	2.8 ± 0.2	1.5 ± 0.1	8.8 ± 0.5	24 ± 1.9	2.8 ± 0.2	11 ± 0.6
T_1/2_ (h)	0.7 ± 0.2	0.7 ± 0.1	0.7 ± 0.1	1.4 ± 0.2	1.6 ± 0.2	3.0 ± 0.6	3.0 ± 1.0
CL (l/h)	0.56 ± 0.20	0.37 ± 0.04	0.68 ± 0.18				
Vd (l/kg)	29 ± 7	20 ± 2	35 ± 7				
F_app_ (%)				43 ± 15	120 ± 40	14 ± 5	54 ± 18

Abbreviations**:** IV = intravenous, IP = intraperitoneal, PO = orally, C_max_ = maximum plasma drug concentration, T_max_ = time to reach maximum plasma concentration, AUC = area under the plasma concentration curve, T_1/2_ = elimination half-life, CL = clearance, Vd = apparent volume of distribution, F_app = _apparent absolute bioavalability.

As shown in Fig. 7, benzavir-2 displayed a rapid uptake from all administration routes. Importantly, benzavir-2 exhibited favourable oral bioavailability properties. However, in an *in vivo* efficacy situation a rapid systemic uptake and relatively fast elimination is not attractive, leading to repetitive administrations to secure a systemic exposure over time. Therefore, we explored new formulation alternatives with a more controlled release of benzavir-2. One promising oral administration alternative is drug-containing PB pellets^18^. Prior to assessing the systemic exposure of benzavir-2 we assessed whether the taste of benzavir-2 would limit the ingestion of the PB pellets. No taste aversion could be detected even with PB pellets containing 100 mg/kg benzavir-2 (data not shown). Feeding mice with benzavir-2 containing PB pellets (60 mg/kg/pellet) lead to high systemic exposure, and importantly, no negative clinical sign such as weight loss or behavioural effect could be observed, indicating that relatively high systemic concentrations of benzavir-2 was well-tolerated in mice over several days. Moreover, the plasma levels of benzavir-2 were well above the EC_50_ value in cell culture (0.6 μM), resulting in a large concentration range for further determination of the antiviral activity in *in vivo* efficacy studies.

To summarize, our data demonstrate potent antiviral activity of benzavir-2 against the rRVFVΔNSs::Katushka infection and the production of new infectious viruses together with a promising pre-clinical PK profile. Moreover, oral administration of benzavir-2 containing PB pellets potentiated a high systemic exposure with no associated negative effect on the mice. This data support further exploration of the antiviral activity of benzavir-2 in *in vivo* efficacy models.

## Methods

### Viruses and vector

Recombinant RVFV expressing the far-red fluorescent protein Katushka instead of the deleted NSs protein (rRVFVΔNSs::Katushka) was used in this study. Generation of rRVFVΔNSs::Katushka has previously been described^27^.

### Compounds

Benzavir-1, 17d, 17e and 35f (Fig. 1) were synthesized as previously described^14^. Benzavir-2 was synthesized according to an improved protocol described in the Supplementary Material. All compounds were >95% pure according to reversed phase HPLC UV traces at 214 nm. Analytical data for all compounds were in agreement with those reported previously^14^. Stock solutions (10 mM) were prepared in dimethylsulfoxide (DMSO) and stored dark at room temperature.

### Cell and culture conditions

Human lung adenocarcinoma basal epithelial cells, A549, were cultured in cell culture medium (Dulbecco’s Modified Eagle’s Medium [DMEM], Sigma-Aldrich, St. Louis, MO) containing 0.75 g NaHCO_3_/L, 20 mM HEPES (4-[2-hydroxyethyl]- 1 piperazineethanesulfonic acid) (EuroClone, Milan, Italy), penicillin G (100 IU/mL), and streptomycin sulfate (100 μg/ mL) combined (1× PEST, Gibco, Carlsbad, CA), and 5% fetal bovine serum (FBS, Gibco) at 37 °C. For virus infection, cell maintenance medium was used containing the same components, except at a lower FBS concentration (2%).

### Fluorescent cell foci assay

To determine the EC_50_ value of the compounds, the fluorescent intensity of individual infectious cell foci was quantified in a dose-dependent manner for all compounds, basically as described previously^27^. Briefly, A549 cells (1 × 10^4^ /well) were seeded in 96-well black plates with transparent bottoms (Greiner Bio-One International) on the day before infection. On the day of infection, compounds were serially diluted in two-fold steps from 25 μM to 0.097 μM and mixed with 1,000 plaque forming units of rRVFVΔNSs::Katushka virus in a total volume of 100 μL DMEM containing 2% FBS, multiplicity of infection (MOI) = 0.1. The final concentration of DMSO in the assay was 0.25%. The growth medium was removed from the wells and 100 μL of compound and virus mixture was added to the cells for each compound concentration. Plates were incubated at 37 °C in 5% CO_2_ for 16 h. Later, the medium was removed and cells were fixed with 3% paraformaldehyde (PFA) for 1 h and washed with phosphate-buffered saline (PBS). Finally, 300 μL PBS was added to each well and the plate was analyzed in the Trophos plate runner HD (Trophos, Roche Group) to count the number of virus infected cells/well, by identifying all individual cells expressing the far-red fluorescent protein Katushka as a measurement of infection^27^. The EC_50_ value was calculated with non-linear regression analysis with a variable slope using GraphPad Prism software version 7.0a (GraphPad Software La Jolla, CA, USA). All laboratory work with rRVFVΔNSs::Katushka virus was performed under biosafety level-2 conditions as approved by the Swedish Work Environment Authority.

### Toxicity of benzavir-2 and its analogs on host cells

The toxicity assessment is decribed in previous publications^13–15^. In brief, XTT-based *In Vitro* Toxicology Assay Kit (Sigma-Aldrich) was used to measure the percentage of viable cells in the precense of compound. The percentage of viable cells after 24 h incubation with 30 or 60 μM benzavir-2 on A549 cells, the cell line used in this study, was 97% for both concentrations^14^.

### Quantitative real time polymerase chain reaction (PCR) and detection of progeny virus

One day before infection, 7 × 10^4^ A549 cells/well were seeded in 24-well plates (Nunc). On the day of infection, growth medium was removed and cells were infected with rRVFVΔNSs::Katushka (MOI = 1). Simultaneously, two-fold serially diluted benzavir-2 ranging from 20 μM to 0.156 μM were added to the cells and incubated for 1 h at 37 °C. The final concentration of DMSO in the assay was 0.25%. After incubation, virus inoculum and benzavir-2 mixture was removed and fresh DMEM (2% FBS) containing same concentration of benzavir-2 were added to the corresponding well and incubated for another 15 h at 37 °C in 5% CO_2_. After incubation, the supernatant medium was collected for each concentration and saved at 4 °C for later use to measure the production of progeny particles. The cells were washed with PBS and lysed with Proteinase K and the total cellular RNA was extracted by using LIAISON^®^ Ixt Viral NA Extraction kit (DiaSorin, Dublin, Ireland) according to the manufacturer’s instructions. RVFV RNA was detected using a previously described primer and Taqman probe from the L-segment which encodes the RVFV RNA dependent RNA polymerase^28^ with the QuantiTect one-step Probe RT-PCR kit (Qiagen). In the subsequent PCR reactions, the actin mRNA was detected with Hs_ACTB_2_SG QuantiTect Primer Assay, QT01680476 (Qiagen) and KAPA SYBR FAST One-Step qRT-PCR Kits (Kapa Biosystems, USA) and the Ct values of RVFV RNA was normalized to β-actin mRNA. ABI StepOnePlus™ Real-Time PCR System (Applied Biosystems, California, USA) was used to quantify the RVFV RNA by relative quantification^29^.

To detect the production of progeny viruses, 1 × 10^4^ A549 cells/well were seeded in 96-well black plates with transparent bottom (Greiner Bio-One International GmbH). Next day, growth medium was removed and the previously saved medium from different concentrations of benzavir-2 (100 μL/well) were added to the cells, and plates were incubated for 16 h at 37 °C. After incubation, medium was removed and cells were fixed with 3% PFA for 1 h and washed with PBS. The number of infected cells per well was counted using the Trophos plate runner HD as described above.

### Time-of-addition assay

The fluorescent cell foci assay was used for time-of-addition assay, as previously described. Briefly, 1 × 10^4^ A549 cells per well were seeded in 96-well black plates with transparent bottom one day prior to experiment. Two hours prior to infection, the media in quadruplicate wells was replaced with 20 μM benzavir-2 in DMEM containing 2% FBS. Then, all the wells were infected with 0.5 MOI of rRVFVΔNSs::Katushka for 30 min at 37 °C. In the 0 h to 2 h wells, 20 μM benzavir-2 was added together with the virus for 30 min that was later removed and replaced with media containing 20 μM benzavir-2 for the remaining 1.5 h. Thirty minutes post infection, the virus was removed from all the wells and 20 μM benzavir-2 was added every second hour in 2 h pulses (0 h to 2 h, 2 h to 4 h, 4 h to 6 h, 6 h to 8 h). In one sample (0 h to 8 h), 20 μM benzavir-2 was present until read-out. Eight hours post infection the infection was assessed by fluorescent cell foci assay as previously described.

### *In vivo* formulation of benzavir-2

In order to increase the solubility of benzavir-2 and to reach acceptable concentrations *in vivo*, a panel of excipients was assessed. From these, two formulation alternatives were selected. Formulation 1: A 1:1 mix of dimethyl sulfoxide (DMSO) and ethanol with exactly one equivalent of NaOH, to form the Na-salt of benzavir-2, was prepared. The solution was added to a vial with corresponding amount of benzavir-2 and was mixed thoroughly using vortex and sonication until a clear solution was developed. The clear solution was diluted 10 times in MQ H_2_O and pH was determined to 7.6–7.8. No precipitation was observed over a period of several days when stored at room temperature. The clear formulation was stored at room temperature until use. Formulation 2: 20 mg/g of benzavir-2 powder was dissolved in a vehicle consisting of 5% (w/w) Tween 80 (Sigma-Aldrich, Missouri, USA) in N-methyl-2-pyrrolidone (Sigma-Aldrich, Missouri, USA). The clear solution was diluted 15 times in 2.6% Glycerol (Sigma-Aldrich, Missouri, USA) in MQ H_2_O prior to administration. The choice of formulation did not influence the pharmacokinetic properties of benzavir-2 in mouse (data not shown).

### *In vivo* pharmacokinetics

Nine week female BALB/C mice (Scanbur A/S, Karlslunde, Denmark) were administered either intraperitoneally, intravenously (iv, tail vein injection at 1 ml/kg) or orally (po) using oral gavage. Several different doses were given via the different routes of administration, iv 1–10 mg/kg, ip and po 1–40 mg/kg. Four mice per administration group were divided in two subgroups with four to five blood samplings respectively. The blood samples were collected from the tail vein at 5, 15, 30, 45, 60, 120, 240, 360 and 480 min after administration. Plasma was collected using Microvette® CB 300 EDTA tubes (Sarstedt Inc.), according to manufacturer’s instruction. All samples were stored at −20 °C until quantitative analysis. As analytical control, plasma was collected from mice that did not receive benzavir-2. Warfarin (0.1 µM) was used as an internal standard. All animal experiments in the study were ethically approved (A31–14) by the Animal Research Ethics Committee of Umeå University (Umeå, Sweden). All experiments were performed in accordance with relevant guidelines and regulations.

### Preparation of peanut butter (PB) pellets containing 60 mg/kg benzavir-2

The preparation was performed as previously described with few exceptions^18^. In brief, peanut butter (Skippy® Creamy) was heated to 55 °C in water bath and the corresponding amount of benzavir-2 was added to achieve 60 mg/kg in mouse. The PB and benzavir-2 powder mixture was mixed by hand with spatula for homogeneous suspension. For the habituation procedure of PB to mice, no benzavir-2 was added to PB. The warm PB mixture was carefully dripped into a pellet mold (Corticosterone Pellet Mold, Prod No. 106A, Ted Pella, Inc., Redding, CA) and any eventual air pockets were removed with spatula. The molds were frozen at −80 °C for minimum 1 h. The frozen pellets were carefully removed and stored at −80 °C until use.

### Habituation of PB to mice and taste aversion

PB pellets were presented to the mice to assure rapid ingestion of the total PB pellet, as described previously with few exceptions^18^. Briefly, frozen PB pellets were added in singlets to square weighing dishes (WVR international Cat. No. 611–9178) and were left in the cage (one mouse per cage). This was performed for 5–7 consecutive days until the PB pellets were rapidly and totally ingested. Additionally, PB pellets containing 30, 60 and 100 mg/kg benzavir-2 were administered to mice for taste aversion studies. No difference in taste aversion could be observed for any of the benzavir-2 concentrations.

### Benzavir-2 administration in PB pellets and blood collection

The mice were weighed on the day prior to experiment and the mean body weight per group was calculated. The body weight determined the amount of benzavir-2 to be added to reach 60 mg/kg per 100 mg pellet. The PB pellets were administered in singlets on square weighing dishes (WVR international Cat. No. 611–9178) in the morning and were left in the cage (one mouse per cage) until all PB was ingested. Three respectively 6 h after PB pellet administration blood was withdrawn from retro-orbital puncture and plasma was collected using Microvette® CB 300 EDTA tubes (Sarstedt Inc.), according to the manufacturer’s instructions.

### Quantitative liquid chromatography tandem mass spectrometry (LC-MS/MS) analysis of benzavir-2 *in vitro* and *in vivo* samples

Plasma samples were analyzed by LC-MS/MS on a Waters Acquity UPLC coupled to either a Waters XEVO TQ or a Sciex QTRAP6500 triple quadrupole mass spectrometer. Analytical separation was performed using mobile phases (A) 0.1% formic acid with 5% MeCN, (B) 0.1% formic acid in MeCN and column BEH C18 1.7 μm 2 × 50 mm from Waters. Analytical standard curve was created using blank matrix from respective assay, e.g. plasma from protein binding and *in vivo* analysis spiked with benzavir-2. Proteins from plasma samples were removed by addition of ice-cold methanol (1:4), from liver microsome and hepatocyte incubations using acetonitrile (1:3). All analysis was performed in 96-well format, plates were sealed and centrifuged at 3500 rpm and either analyzed directly or frozen until LC-MS/MS analysis.

### Calculations of pharmacokinetic properties

Calculation of non-compartmental PK was performed utilizing Microsoft Excel and Graphpad Prism 7. The measured concentrations was normalized to a selected dose, e.g. iv 2 and 4 mg/kg was divided with 2 and 4, respectively. The ip and po doses were normalized to 10 mg/kg. The errors (SEM) indicated stemmed from fitting in Graphpad or combined errors where appropriate. *In vitro* predictions of absorption, distribution, metabolism, and excretion (ADME) were performed as described in^16,17,30^.

### Data availability

The datasets generated during and/or analysed during the current study are available from the corresponding author on reasonable request.

## Electronic supplementary material

Supplementary material
