# Supplementary material for: Anti-Rift Valley fever virus activity in vitro, pre-clinical pharmacokinetics and oral bioavailability of benzavir-2, a broad-acting antiviral compound
Source: Sci Rep. 2018 Jan 31;8:1925. doi: 10.1038/s41598-018-20362-9 (PMC5792431; doi:10.1038/s41598-018-20362-9)
Supplement: Supplementary file 1 — Supplementary material [file 41598_2018_20362_MOESM1_ESM.doc]

**Anti-Rift Valley fever virus activity *in vitro*, pre-clinical pharmacokinetics and oral bioavailability of benzavir-2, a broad-acting antiviral compound**

Md. Koushikul Islam^1^, Mårten Strand^2^, Michael Saleeb^3^, Richard Svensson^4, 5^, Pawel Baranczewski^4, 5^, Per Artursson ^4, 5, 6^, Göran Wadell^2^*,* Clas Ahlm^1^, Mikael Elofsson^3^, Magnus Evander^2*^

Department of Clinical Microbiology, Infectious Diseases, Umeå University, Umeå Sweden^1^; Department of Clinical Microbiology, Virology, Umeå University, Umeå Sweden^2^; Department of Chemistry, Umeå University, Umeå Sweden^c3^; Uppsala Drug Optimization and Pharmaceutical Profiling Platform (UDOPP), Department of Pharmacy, Uppsala University, Uppsala, Sweden^4^. SciLifeLab Drug Discovery and Development Platform, ADME of Therapeutics, Department of Pharmacy, Uppsala University^5^, Department of Drug Delivery, Institute of Pharmacy, Uppsala University^6^

**SUPPLEMENTAL MATERIAL**

**Experimental section**

GENERAL CHEMISTRY

All reactions were carried out under inert atmosphere (N_2_ gas). Chemicals and reagents were purchased from Aldrich, Alfa Aesar, AK Scientific, Matrix Scientific or Apollo Scientific. Organic solvents were dried using the dry solvent system (Glass Contour Solvent Systems, SG Water USA) except CH_3_OH, which was dried over molecular sieve 3Å. Flash chromatography was performed on Biotage Isolera One using appropriate SNAP Cartridge KP-Sil and UV absorbance at 254 nm. TLC was performed on Silica gel 60 F_254_ (Merck) with detection by UV light unless staining solution is mentioned. The NMR spectra were recorded at 298 K on Bruker-DRX 400 MHz and 600 MHz using the residual peak of the solvent DMSO-*d*_6_ (δ_H_ 2.50 ppm) or CDCl_3_ (δ_H_ 7.26 ppm) as internal standard for ^1^H, and DMSO-*d*_6_ (δc 39.50 ppm) and CDCl_3_ (δ_c_ 77.16 ppm) as internal standard for ^13^C. LC-MS data were recorded by detecting positive/negative ion (EC^+^/EC^-^) with an electrospray Water Micromass ZG 2000 instrument using XTerra MS C_18_ (5 μm, 19x50 mm column) and H_2_O/CH_3_CN (0.2% HCOOH) as the eluent system, or with Agilent 1290 infinity – 6150 Quadrupole using YMC Triart C_18_ (1.9 μm, 20x50 mm column) and H_2_O/CH_3_CN (0.1% HCOOH) as the eluent system. All compounds were >95% pure according to LC-MS chromatograms using UV detection at 214 nm.

SYNTHESIS

**Synthesis of ethyl 2-(4,5-difluoro-2-nitrobenzamido)benzoate - I**

4,5-Difluoro-2-nitrobenzoic acid (3 g, 14.85 mmol) was dissolved in DCM (23 mL) and kept at 0 °C under N_2_ before oxalyl chloride (5.2 mL, 4 eq) was added dropwise followed by addition of catalytic amount of anhydrous DMF (0.2 ml, 0.2 eq). The mixture was stirred for 30 min at 0 °C and for additional 2 h at room temperature. The acid chloride formation was monitored by heating an aliquot in CH_3_OH and check LC-MS for methyl ester formation. The reaction mixture was concentrated under vacuum and co-evaporated with DCM repeatedly. The crude was dissolved in DCM (75 ml) at 0 °C and dry pyridine (2.4 mL, 2.0 eq) was added followed by addition of ethyl anthranliate (1.96 mL, 0.9 eq) and the mixture was stirred at room temperature overnight. The reaction mixture was diluted with DCM and washed twice with HCl (1M), and once with saturated solution of NaHCO_3_, dried over MgSO4 and concentrated under vacuum. The product was purified with chromatography using DCM/Heptane/CH_3_OH 6:4:0.05 as eluent and product of Rf=0.36 (DCM/Heptane/CH_3_OH 6:4:0.05) was collected to afford ethyl 2-(4,5-difluoro-2-nitrobenzamido)benzoate – I (4.6 g, 89% yield)

**Synthesis of ethyl 2-(2-amino-4,5-difluorobenzamido)benzoate - II**

Ethyl 2-(4,5-difluoro-2-nitrobenzamido)benzoate I (4.45 g, 12.7 mmol) and ammonium formate (76.2 mmol, 6 eq) were dissolved in dry CH_3_OH (77 mL). Pd/C 10% (0.2 eq) was added and the mixture was refluxed for 2h and monitored with TLC. The reaction was completed within 2-3 h. The mixture was filtered over a pad of Celite and concentrated under vacuum. NB: The product is unstable and should be protected from direct light and kept under inert condition. The product was crystalized from CH_3_OH under N_2_ and darkness. The crystal was allowed to grow overnight under N_2_, collected and washed with ice-cold CH_3_OH to afford ethyl 2-(2-amino-4,5 difluorobenzamido)benzoate – II (88% yield).

**Synthesis of ethyl 2-(4,5-difluoro-2-(2-fluorobenzamido)benzamido)benzoate - III**

Ethyl 2-(2-amino-4,5 difluorobenzamido)benzoate - II (3.6 g, 11.3 mmol) was dissolved in dry DCM (102 mL) and pyridine (3 eq) was added followed by addition of 2-Flourbenzoyl chloride (1.5 eq) at 0 °C and the mixture was stirred at room temperature overnight. The reaction was monitored with TLC ((DCM/CH_3_OH 1%)/Heptane 1:1, Rf= 0.34). The reaction mixture was diluted with DCM and washed with HCl (1M), saturated solution of NaHCO_3_ and concentrated under vacuum. The product was purified on silica gel using DCM as eluent to afford ethyl 2-(4,5-difluoro-2-(2-fluorobenzamido)benzamido)benzoate – III (5.2 g, quantitative yield).

**Synthesis of 2-(4,5-difluoro-2-(2-fluorobenzamido)benzamido)benzoic acid – IV**

Ethyl 2-(4,5-difluoro-2-(2-fluorobenzamido)benzamido)benzoate - III (5.2 g, 11.7 mmol) was dissolved in DMSO (220 mL) and NaOH (2M solution, 117 mL, 20 eq) was added and the mixture was stirred at room temperature for 4 h. The mixture was acidified with HCl (6M) and the precipitate was filtered off and washed thoroughly with H_2_O, diethyl ether and DCM. The product was dried under high vacuum to afford 2-(4,5-difluoro-2-(2-fluorobenzamido)benzamido)benzoic acid - IV (3.5 g, 72%) >95% pure.

**In vitro ADME and analytical methods**

Analytical procedures

All assays and in vivo samples were analyzed by ultra performance liquid chromatography coupled to mass spectrometry (LC–MS/MS). The following systems was used: Waters Acquity UPLC coupled to either a Waters XEVO TQ mass spectrometer or a Sciex QTRAP6500 (electrospray ionization, ESI(-)). For chromatographic separation a general gradient was used (1% mobile phase B to 90% over 2 min total run) on a C18 BEH 1.7 μm column 2 × 50 mm (Waters Corp.). The mobile phase A consisted of 5% acetonitrile 0.1% formic acid and the mobile phase B 100% acetonitrile 0.1% formic acid. The flow rate was 0.5 ml/min. For analysis of plasma, solubility, LogD or Caco-2 permeability a standard curve between 1 and 10 000 nM was prepared. No detailed method validation was performed and an accurate limit of detection is therefore not known. The method sensitivity was however very good with an estimated limit of detection <1 nM. Analysis of metabolic stability does not require a standard curve since it is quantified on a relative basis.

Plasma protein binding

We utilized Rapid Equilibrium Dialysis (RED) device inserts which allow for short dialysis times (2–4 h) as compared to traditional methods (>8 h) and minimal drug consumption in a 96-well format. Pooled human plasma (EDTA) was provided by Uppsala Academic Hospital and was collected from two male and two female donors (non-smoking). In brief, 0.2 mL of the plasma test solution with 10 μM final compound concentration was transferred to the membrane tube in the RED insert. Then 0.35 mL isotonic phosphate buffer pH 7.4 was added to the other side of the membrane. The 96-well base plate was then sealed with an adhesive plastic film (Scotch Pad) to prevent evaporation. The plate was incubated with rapid rotation (≈900 rpm) on a Kisker rotational incubator at 37 °C for 4 h to achieve equilibrium. A stability test of the test solution was also prepared (to allow detection of drug degradation). For this, 100 μL of the plasma test solution (in a plastic vial or on a sealed plate) was incubated at 37 °C for 4 h (as long as the dialysis time). The plasma test solution was frozen directly after the administration to prevent degradation. After incubation, the contents of each plasma and buffer compartment were removed and immediately frozen until analysis. Prior to LC–MS/MS analysis the samples were mixed with equal volumes of control buffer or plasma as appropriate to maintain matrix similarity for analysis. Plasma proteins were then precipitated by the addition of methanol (1:4) containing Warfarin as analytical internal standard. The plate was then sealed, centrifuged and the supernatant was analyzed by LC-MS/MS.

Thermodynamic solubility

Studies on from solid compound (free acid) solubility were performed in PBS (pH 7.4). and in an HCl-adjusted PBS to low pH, approximately pH 1.2. The study was performed by weighing approximately 0.5 mg of compound in a HPLC vial and addition of buffer to produce a saturated solution. The vial was sealed and shaken for 24h at 37 °C. After the incubation an aliquot of the solution was transferred to a glass vial insert and centrifuged at 10 000 × g, 37 °C, for 20 min. The supernatant was analyzed by LC-MS/MS as described above.

LogD pH 7.4

In vitro lipophilicity measurements utilized a miniaturized shake-flask method in HPLC vials. In brief, 1.2 µl compound was added to a vial, then 1 ml phosphate buffer followed by 0.2 ml octanol. The buffer and octanol was previously saturated with a small amount octanol and buffer, respectively. The vials were then sealed and shaken for 2h in room temperature. The phases were then separated by let standing (in dark) during 48h. After separation the octanol phase was carefully removed and both phases quantified by LC-MS/MS.

Caco-2 permeability

The Caco-2 study was performed in accordance with published protocols^1^. Caco-2 cell monolayers (passage 94–105) were grown on permeable filter support and used for transport study on day 21 after seeding. Prior to the experiment a drug solution of 10 μM was prepared and warmed to 37 °C. The Caco-2 filters were washed with pre-warmed Hank's balance salt measurement (HBSS) prior to the experiment, and thereafter the experiment was started by applying the donor solution on the apical side. The transport experiments were carried out at pH 7.4 in both the apical and basolateral chambers. The experiments were performed at 37 °C and with a stirring rate of 500 rpm. The receiver compartment was sampled at 15, 30 and 60 min, and at 60 min also a final sample from the donor chamber was taken in order to calculate the mass balance of the compound. Directly after the termination of the experiment the filter inserts were washed with pre-warmed HBSS and the membrane integrity was checked. This was performed by transepithelial electrical resistance (TEER) measurement and by measurement of Mannitol permeability, which is a paracellular marker used for integrity measurements.

Metabolic stability

The microsomal metabolic stability assay utilized pooled human or animal species, liver microsomes (LM) with supplemented cofactor (NADPH) to facilitate CYP reactivity against target compounds. Human hepatocytes (HHep) were freshly prepared at UDOPP (liver from surgery at the academic hospital in Uppsala on the same day). For incubation, target compound (1 μM in the incubation) and LM (0.5 mg/ml) or hepatocytes (0.5 × 10^6^ cells/ml) were diluted in 0.1 M phosphate buffer pH 7.4 (LM) or Williams medium E (HHep). The incubation volume was 700 μL. The reaction with LM was initiated with addition of NADPH (1 mM incubation concentration) in buffer or by the addition of cells with HHep. The incubation times were 0, 5, 15, 40 min (in duplicate for LM) or 0, 10, 30, 60 min (in duplicate for HHep). At the indicated time points an aliquot (100 µl) was transferred to a 96-well plate containing 100 µl acetonitrile with Warfarin as the analytical internal standard. The plate was then sealed, centrifuged and frozen at −20 °C until LC–MS/MS analysis.

**REFERENCES**

1 Hubatsch, I., Ragnarsson, E. G. & Artursson, P. Determination of drug permeability and prediction of drug absorption in Caco-2 monolayers*. Nature protoco*l**s** 2, 2111-2119, doi:10.1038/nprot.2007.303 (2007).
